# Synthesis and Reactivity of [1,2,4]Triazolo-annelated Quinazolines

**DOI:** 10.3390/molecules15107016

**Published:** 2010-10-12

**Authors:** Rashad A. Al-Salahi

**Keywords:** triazolo[1,5-*a*]quinazolines, thionation, alkylation, chlorination, tetracyclic systems

## Abstract

This paper reports the synthesis of phenyl-substituted 2-alkoxy(methylsulfanyl)-1,2,4-triazolo[1,5-*a*]quinazolines starting from *N*-cyanoimidocarbonates and substituted hydrazinobenzoic acids as building blocks. Thionation or chlorination of the inherent lactam moiety in the target compounds followed by treatment with multifunctional nucleophiles provided access to a variety of derivatives.

## 1. Introduction

Triazolo-annelated quinazolines are known to constitute a pharmacologically interesting class of compounds. For instance, the novel compound **Ia** is effective adenosine antagonist whereas the related compound **Ib** was found to be benzodiazepine receptor antagonist [1,2,3]. The recently reported 1,2,4-triazoloquinazolines of type **II** were also found to exhibit promising antihistaminic activity against histamine induced bronchospasms and showed negligible sedation, compared to chlorpheniramine maleate, and could therefore serve as lead molecules for further modification to obtain a clinically useful class of non-sedative antihistamines [4,5]. Furthermore, some triazoloquinazolines **IIIa **which originated from *N*-cyanoimidocarbonates as synthons, have been described as potent protein kinase inhibitors [6].

In our previous paper on the 1,2,4-triazolo[1,5-a]quinazolines series **IIIb**, the corresponding alkylated derivatives have been proven as excellent agents for controlling the plant growth diseases caused by fungal pathogens, and some chlorinated compounds have shown an interesting affinity towards adenosine receptors [7].

In continuation of our ongoing studies of the chemistry of 1,2,4-triazolo[1,5-*a*]quinazolines, we report herein the synthesis of several phenyl-substituted 2-alkoxy(methylsulfanyl)-1,2,4-triazolo[1,5-*a*]quinazolines and their derivatives.


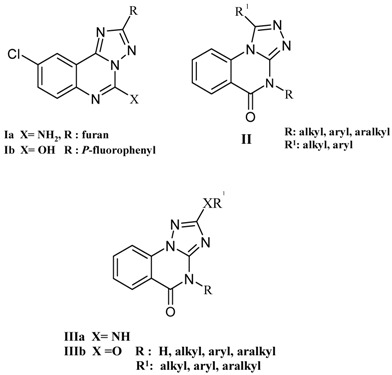


## 2. Results and Discussion

**Scheme 1:**
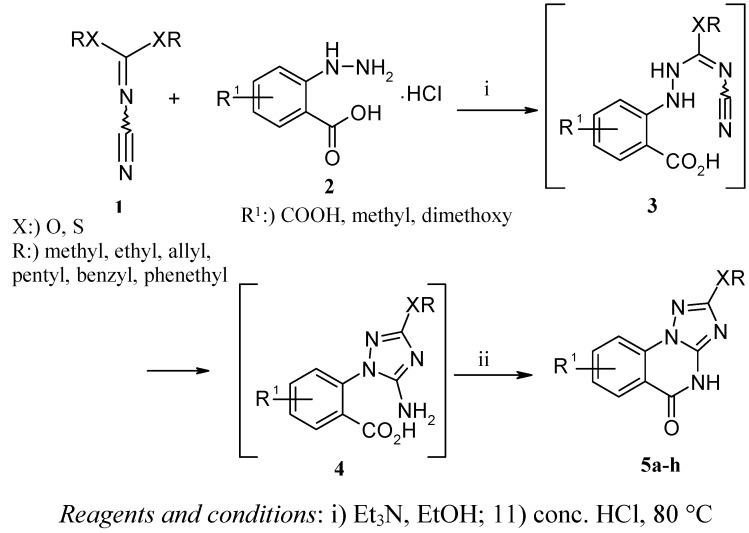
Synthesis of [1,2,4]triazolo[1,5-*a*]quinazolin-5-ones **5a-h**.

The cornerstone of the strategy for the synthesis of our target products was the preparation of compounds **5a-h **(Scheme 1, Table 1). The first step, the preparation of several dialkyl *N*-cyanoimido-carbonates **1** from equimolar amounts of cyanogen bromide and the corresponding alcohol was reported previously [8]. In addition, it has been found that, the reaction of cyanamide with carbon disulfide in the presence of KOH followed by the alkylation with methyl iodide gives dimethyl *N*-cyanoimidodithiocarbonate [9].

**Table 1:** Prepared compounds **5-9**.

Compounds	R	R^1^	R^2^	X
**5a**	CH_3_	COOH	-	O
**5b**	CH_3_CH_2_-	COOH	-	O
**5c**	CH_3_CH_2_CH_2_CH_2_CH_2_-	CH_3_	-	O
**5d**	CH_2_=CHCH_2_-	CH_3_	-	O
**5e**	C_6_H_5_CH_2_-	di-OCH_3_	-	O
**5f**	C_6_H_5_CH_2_CH_2_-	di-OCH_3_	-	O
**5g**	CH_3_	CH_3_	-	S
**5h**	CH_3_	di-OCH_3_	-	S
**6a**	CH_3_	CH_3_	C_6_H_5_CH_2_CH_2_-	S
**6b**	C_6_H_5_CH_2_-	di-OCH_3_	CH_2_=CHCH_2_-	O
**6c**	CH_3_	COOH	CH_3_CH_2_-	O
**6d**	CH_2_=CHCH_2_-	CH_3_	C_6_H_5_CH_2_-	O
**7a**	CH_3_	CH_3_	-	S
**7b**	CH_2_=CHCH_2_-	CH_3_	-	O
**7c**	C_6_H_5_CH_2_-	di-OCH_3_	-	O
**7d**	C_6_H_5_CH_2_CH_2_-	di-OCH_3_	-	O
**8a**	CH_3_	di-OCH_3_	-	S
**8b**	CH_2_=CHCH_2_-	CH_3_	-	O
**8c**	C_6_H_5_CH_2_-	di-OCH_3_	-	O
**8d**	C_6_H_5_CH_2_CH_2_-	di-OCH_3_	-	O
**9a**	CH_3_CH_2_-	COOH	-	O
**9b**	CH_3_CH_2_CH_2_CH_2_CH_2_-	CH_3_	-	O
**9c**	C_6_H_5_CH_2_-	di-OCH_3_	-	O
**9d**	CH_2_=CHCH_2_-	CH_3_	-	O
**9e**	C_6_H_5_CH_2_CH_2_-	di-OCH_3_	-	O
**9f**	CH_3_	CH_3_	-	S

Diazotization of the corresponding anthranilic acids [10] followed by the reduction with sulphur dioxide afforded the substituted 2-hydrazinobenzoic acids **2**. Based on the high reactivity of *N*-cyanoimidocarbonates towards hydrazines to produce 1,2,4-triazole derivatives [11,12,13], reaction of **1** with **2** in ethanol in the presence of triethylamine under ice cooling analogously provided the intermediate 1,2,4-triazole derivatives **4**, which upon treatment with hydrochoric acid produced the target [1,2,4]triazolo[1,5-*a*]quinazolin-5-ones **5a-h **in 50-68% yield [14]. The structures of the novel compounds **5a-h **have been established on the basis of their IR, ^1^H-NMR and ^13^C-NMR spectra and microanalysis. 

The IR spectra of compounds **5a-h** are characterized by a strong (C=O)-stretching band at 1,685-1,712 cm^−1^. 

**Scheme 2:**
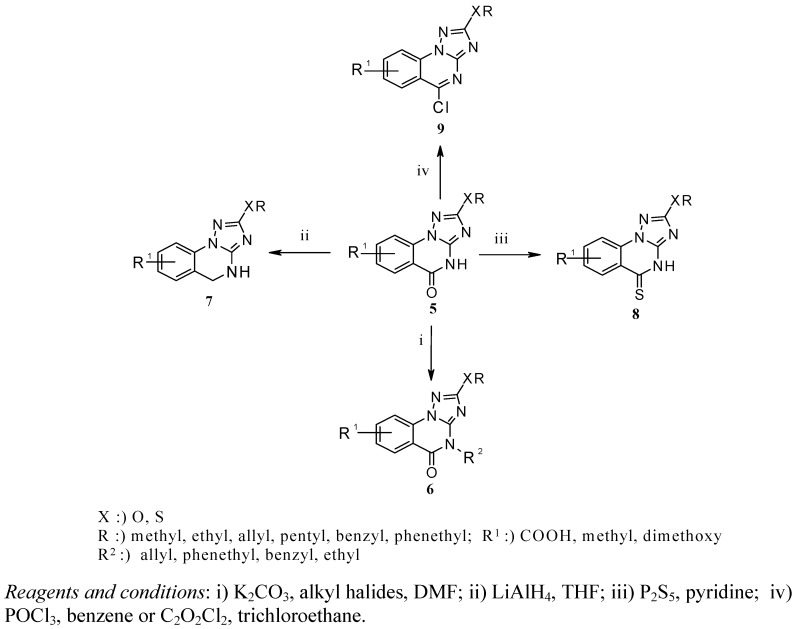
Synthesis of compounds **6-9**.

Alkylation of the lactam functionality may occur at the *N-* or (and) *O*-atom, giving rise to the formation of *N*-alkyllactams or (and) cyclic imido esters [15,16,17]. Regioselective *N*-alkylation has been well documented in the literature [18,19]. Accordingly, when the [1,2,4]triazolo[1,5-*a*]quinazolin-5-ones **5** were allowed to react with alkyl halides in a molar ratio of 1:1.5 in absolute dimethyl formamide at room temperature in the presence of potassium carbonate, the corresponding 4-alkyl[1,2,4]triazolo[1,5-*a*]quinazolin-5-ones **6a-d** resulted in 62-85% yield (Scheme 2, Table 1) [18].

The products **6a-d **were obtained as colored solid compounds and their IR spectra display a strong (C=O) absorption band at 1,670-1,682 cm^−1^. Treatment of compounds **5** with lithium aluminum hydride in absolute tetrahydrofuran at room temperature furnished the expected 4,5-dihydro[1,2,4]triazolo[1,5-*a*]quinazolines **7a-d** in 55-70% yield [20]. The compounds **7a-d** were obtained as colorless solids after column chromatography and their structures were verified by elemental analyses and spectral (NMR, MS and IR) data. The IR revealed the disappearance of the (C=O) absorption band at 1,685-1,712 cm^−1^ (previously found in compounds **5**) and confirmed the formation of the products **7**. When equimolar amounts of [1,2,4]triazolo[1,5-*a*]quinazolin-5-ones **5 **and phosphorus pentasulfide were allowed to react in absolute pyridine under reflux for 2 h, the desired 2-alkoxy(methylsulfanyl)-4*H*-[1,2,4]triazolo[1,5-*a*]quinazolin-5-thiones **8a-d** could be isolated as yellow solids in excellent yields of 89-95% [21]. The IR spectra of compounds **8a-d **displayed a weak (C=S) absorption band at around 1,249-1,268 cm^−1 ^and the ^13^C-NMR spectra were characterized by a (C=S) resonance at 184.91-186.62 ppm.

Conversion of [1,2,4]triazoloquinazolin-5-ones **5** into 5-chloro-[1,2,4]triazolo[1,5-*a*]quinazolines **9a-f** has been successfully achieved by chlorination with either oxalyl chloride in boiling 1,1,2-trichloroethane for 19 h [14] or with phosphorus oxychloride in boiling benzene for 2 h, followed by trituration with a saturated aqueous solution of potassium carbonate [22]. Although both methods gave acceptable yields, the reaction of **5** with phosphorus oxychloride is more advantageous with regard to short reaction time and higher yields. The formation of **9** was accompanied by the gradual disappearance of the characteristic (C=O) band of **5** at 1,685-1,712 cm^−1^.

**Scheme 3:**
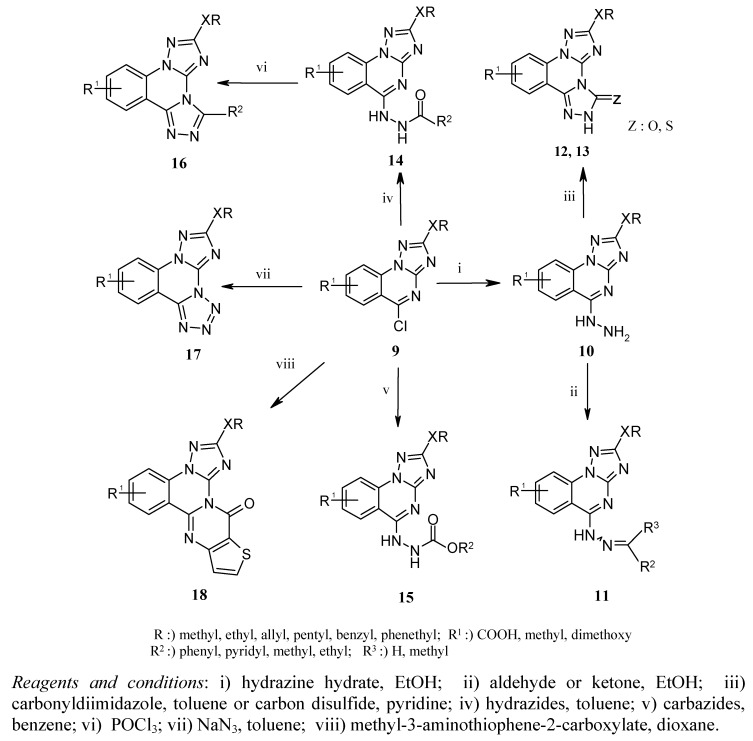
Synthesis of compounds **10-18**.

As outlined in Scheme 3, hydrazinolysis of **9** in refluxing ethanol led to the corresponding [1,2,4]triazolo[1,5-*a*]quinazolin-5-yl-hydrazines **10a-d** in good yields of 60-78% [23], which upon treatment with an equimolar amount of aldehyde or ketone furnished the respective hydrazones **11a-d **in 68-83% yield (Table 2) [24]. The ^1^H-NMR spectra of compounds **10** showed signals of NH_2_, NH at δ 4.65-5.40 and 9.37-9.90 ppm respectively, whereas the structure of the hydrazones **11** was confirmed by disappearance of the signal of NH_2_ group in the ^1^H-NMR spectra. Reaction of **10 **with 1,1^׳^-carbonyldiimidazole in a molar ratio of 1:1.2 in boiling absolute toluene for 3 h provided the hitherto unknown bis[1,2,4]triazolo[1,5-*a*:4,3-*c*]quinazolin-3-ones **12a**,**b **in 49 and 57% yield [25]. Similarly, the corresponding thioxo derivatives **13a**,**b** could be obtained in 56 and 61 % yield from the reaction of **10 **with carbon disulfide in a molar ratio of 1:10 in refluxing pyridine for 2 h [26]. The IR spectra of **12 **display strong (C=O) absorption bands at 1,702 and 1,711 cm^−1^, and the ^13^C-NMR spectra of **13** are characterized by a (C=S) resonance at 185.05 and 185.73 ppm. Replacement of the chlorine in compounds **9 **by different hydrazides occurred smoothly in refluxing toluene to produce the [1,2,4]triazoloquinazolin-5-yl-carbohydrazides **14a**,**b** in 65 and 76% yield [27]. The IR spectra of **14** are characterized by a strong (C=O) absorption band at 1,660, 1,673 and a weak (NH) absorption band at 3,184, 3,207 cm^−1^, respectively. Like the reaction with hydrazides, the corresponding reaction of compounds **9** with carbazides according to literature [27] produced the respective [1,2,4]triazoloquinazolin-5-yl-hydrazine-carboxylic acid esters of type **15a**,**b **in 75 and 80% yield as colorless solids. The IR spectra of **15 **display a strong (C=O) absorption band at 1,708, 1,718 and a weak (NH) absorption band at 3,198, 3,261 cm^−1^.

After having successfully elaborated the synthesis of the carbohydrazides **14**, we became interested in seeing whether these compounds could be cyclo-condensed to the novel bis[1,2,4]triazoloquinazolines of type **16**. In fact when amidrazones **14** were treated with phosphorus oxychloride at refluxing temperature for 2 h, followed by subsequent neutralization with saturated potassium carbonate solution or aqueous ammonia, the desired compounds **16a**,**b **were obtained in 70 and 75% yield [28]. The completion of the internal cyclization was monitored by IR spectroscopy: disappearance of the (C=O) and (NH) absorption bands at 1,660, 1,673 and 3,184, 3,207 cm^−1^ signaled complete conversion of **14** to the tetracyclic compounds **16**. When 5-chloro[1,2,4]triazoloquinazolines **9 **were reacted with sodium azide in a molar ratio of 1:1.2 in absolute dimethyl formamide for 24 h at 90°C, the corresponding 2-alkoxy(methylsulfanyl)-tetrazolo[4,3-*c*][1,2,4]triazolo[1,5-*a*]quinazolines **17a-c **were formed as colorless solids in 51-60% yield (Scheme 3, Table 2) [29].

The aforementioned facile nucleophilic displacement of the chlorine atom in **9 **prompted us to investigate the reaction of **9** with methyl 3-amino-thiophene-2-carboxylate, which theoretically should provide access to the novel pentacyclic compounds of type **18**. Thus, when compounds **9** were reacted with methyl 3-amino-thiophene-2-carboxylate in absolute dioxane in a molar ratio of 1:1.6, followed by addition of sodium hydride, the target compounds **18a**,**b** could be isolated from the reaction mixture in 69 and 81% yield [27]. The IR spectra of compounds **18 **are characterized by (C=O) stretching bands at 1,670 and 1,677 cm^−1^.

**Table 2:** Prepared compounds **10-18**.

Compounds	R	R^1^	R^2^	R^3^	X/Z
**10a**	CH_3_	CH_3_	-	-	S
**10b**	CH_3_ CH_2_-	COOH	-	-	O
**10c**	CH_2_=CHCH_2_-	CH_3_	-	-	O
**10d**	C_6_H_5_CH_2_-	di-OCH_3_	-	-	O
**11a**	CH_3_	CH_3_	CH_3_	CH_3_	S
**11b**	CH_3_	CH_3_	C_6_H_5_	H	S
**11c**	C_6_H_5_CH_2_-	di-OCH_3_	CH_3_	CH_3_	O
**11d**	C_6_H_5_CH_2_CH_2_-	di-OCH_3_	C_6_H_5_	CH_3_	O
**12a**	CH_2_=CHCH_2_-	CH_3_	-	-	O/O
**12b**	C_6_H_5_CH_2_-	di-OCH_3_	-	-	O/O
**13a**	C_6_H_5_CH_2_-	di-OCH_3_	-	-	O/S
**13b**	CH_3_	CH_3_	-	-	S/S
**14a**	CH_3_	CH_3_	C_6_H_5_	-	S
**14b**	CH_3_	CH_3_	C_5_H_4_N	-	S
**15a**	CH_2_=CHCH_2_-	CH_3_	CH_3_CH_2_-	-	O
**15b**	CH_3_	CH_3_	C_6_H_5_CH_2_-	-	S
**16a**	CH_3_	CH_3_	C_6_H_5_	-	S
**16b**	CH_3_	CH_3_	C_5_H_4_N	-	S
**17a**	CH_3_	CH_3_	-	-	S
**17b**	C_6_H_5_CH_2_-	di-OCH_3_	-	-	O
**17c**	C_6_H_5_CH_2_CH_2_-	di-OCH_3_	-	-	O
**18a**	CH_3_CH_2_CH_2_CH_2_CH_2_-	CH_3_	-	-	O
**18b**	C_6_H_5_CH_2_CH_2_-	di-OCH_3_	-	-	O

**Table 3:** Melting points, crystallization solvents, yields, molecular formulae and molecular weights of compounds **5-18**.

Comp. No.	Mp (°C)	Cryst. Solv.	Yield (%)	Molecular Formula. (Mol. Wt)
**5a**	228-230	THF	58	C_11_H_8_N_4_O_4 _(260.21)
**5b**	239-241	THF	64	C_12_H_10_N_4_O_4_ (274.24)
**5c**	254-257	THF	50	C_15_H_18_N_4_O_2_ (286.34)
**5d**	232-234	THF	55	C_13_H_12_N_4_O_2_ (256.27)
**5e**	243-245	THF	65	C_18_H_16_N_4_O_4_ (352.35)
**5f**	265-267	THF	60	C_19_H_18_N_4_O_4 _(366.38)
**5g**	227-229	THF	68	C_11_H_10_N_4_OS (246.29)
**5h**	216-218	THF	62	C_12_H_12_N_4_O_3_S (292.32)
**6a**	180-182	THF	85	C_19_H_18_N_4_OS (350.35)
**6b**	172-174	THF	81	C_21_H_20_N_4_O_4_ (392.42)
**6c**	165-167	THF	62	C_13_H_12_N_4_O_4_ (288.26)
**6d**	202-204	THF	82	C_20_H_18_N_4_O_2_ (346.39)
**7a**	133-135	EtOAc-hexane	60	C_11_H_12_N_4_S (232.31)
**7b**	145-147	EtOAc-hexane	55	C_13_H_14_N_4_O (242.28)
**7c**	158-160	EtOAc-hexane	70	C_18_H_18_N_4_O_3_ (338.37)
**7d**	179-181	EtOAc-hexane	64	C_19_H_20_N_4_O_3 _(352.40)
**8a**	220-222	DMF	90	C_12_H_12_N_4_O_2_S_2_ (308.38)
**8b**	212-214	DMF	95	C_13_H_12_N_4_OS (272.33)
**8c**	253-255	DMF	92	C_18_H_16_N_4_O_3_S (368.42)
**8d**	241-243	DMF	89	C_19_H_18_N_4_O_3_S (382.44)
**9a**	128-130	THF-hexane	90	C_12_H_9_ClN_4_O_3_ (292.68)
**9b**	144-46	THF-hexane	88	C_15_H_17_ClN_4_O (304.78)
**9c**	163-165	THF-hexane	91	C_18_H_15_ClN_4_O_3_ (370.80)
**9d**	132-135	THF-hexane	87	C_13_H_11_ClN_4_O (274.71)
**9e**	157-159	THF-hexane	93	C_19_H_17_ClN_4_O_3_ (384.83)
**9f**	176-178	THF-hexane	86	C_11_H_9_ClN_4_S (264.74)
**10a**	230-232	EtOH	60	C_11_H_12_N_6_S (260.32)
**10b**	215-217	EtOH	69	C_12_H_12_N_6_O_3_ (288.27)
**10c**	223-225	EtOH	71	C_13_H_14_N_6_O (270.30)
**10d**	243-245	EtOH	78	C_18_H_18_N_6_O_3_ (366.38)
**11a**	189-191	EtOH	70	C_14_H_16_N_6_S (300.39)
**11b**	208-210	EtOH	83	C_18_H_16_N_6_S (348.43)
**11c**	198-200	EtOH	73	C_21_H_22_N_6_O_3_ (406.45)
**11d**	213-215	EtOH	68	C_27_H_26_N_6_O_3_ (482.55)
**12a**	219-221	EtOH	49	C_14_H_12_N_6_O_2_ (296.29)
**12b**	230-232	EtOH	57	C_19_H_16_N_6_O_4 _(392.38)
**13a**	248-250	MeOH	61	C_19_H_16_N_6_O_3_S (408.44)
**13b**	225-227	MeOH	56	C_12_H_10_N_6_S_2 _(302.38)
**14a**	178-179	MeOH	65	C_18_H_16_N_6_OS (364.43)
**14b**	149-151	MeOH	76	C_17_H_15_N_7_OS (365.42)
**15a**	127-129	MeOH	75	C_16_H_18_N_6_O_3_ (342.36)
**15b**	191-193	MeOH	80	C_19_H_18_N_6_O_2_S (394.46)
**16a**	186-188	MeOH	75	C_18_H_14_N_6_S (346.42)
**16b**	200-202	MeOH	70	C_17_H_13_N_7_S (347.40)
**17a**	170-172	MeOH	60	C_11_H_9_N_7_S (271.31)
**17b**	224-226	MeOH	54	C_18_H_15_N_7_O_3_ (377.37)
**17c**	206-208	MeOH	51	C_19_H_17_N_7_O_3_ (391.39)
**18a**	242-244	MeOH	81	C_20_H_19_N_5_O_2_S (393.47)
**18b**	231-233	MeOH	69	C_24_H_19_N_5_O_4_S (473.51)

## 3. Experimental

### 3.1. General

Melting points (°C) were determined on open glass capillaries using a Mettler FP 62 apparatus and are uncorrected. Elemental analyses (C, H, N, S) were in full agreement with the proposed structures within ± 0.4% of the theoretical values, and were carried out with a Heraeus CHN-O-Rapid Instrument. The IR (KBr) spectra were recorded on a Shimadzu FT-IR 8300. ^1^H-NMR (400 MHz) and ^13^C-NMR (100 MHz) spectra were recorded on a Bruker AMX 400 spectrometer and chemical shifts are giving in a (ppm) downfield from tetramethylsilane (TMS) as an internal standard, DMSO is used as solvent. Mass spectra were recorded on a Finnigan MAT 311A and on a VG 70-250S (VG Analytical) instrument. Follow up of the reactions and checking the purity of compounds was made by TLC on DC-Mikrokarten polygram SIL G/UV_254, _from the Macherey-Nagel Firm, Duren Thickness: 0.25 m. Column chromatography was conducted on silica gel (ICN Silica 100-200, active 60 Å)

### 3.2. Chemistry

#### 3.2.1. Synthesis of compounds **5a-h**

10 mmol of substituted hydrazinobenzoic acid **2 **was added portionwise to a stirred solution of **1** (10 mmol) in EtOH (20 mL) at 0°C. Afterwards triethylamine (30 mmol) was added dropwise over a period of 30 min. After the addition was complete, the reaction mixture was left to stir overnight at room temperature. Acidification of the mixture was performed by conc. HCl under ice cooling followed by refluxing for 1-3 h. After cooling, the mixture was poured into ice/water, the resulting solid was filtered, washed with water and dried. Recrystallization from THF gave analytically pure colored cpmpounds **5a-h**.

*8-Carboxylic acid-2-methoxy-4H-[1,2,4]triazolo[[1,5-a]]quinazolin-5-one* (**5a**). IR (cm^−1^): ν 1,685, 1,712 (C=O). ^1^H-NMR (DMSO-d_6_): δ 3.19 (s, 1H, OH), 3.99 (s, 3H, OCH_3_), 7.48-8.05 (m, 3H, Ar-H), 13.15 (s, 1H, NH). ^13^C-NMR: 57.16, 114.31, 116.53, 125.58, 128.08, 135.12, 136.18, 147.87, 159.70, 161.83, 168.02. MS, *m/z* (%): 260 (M^+^, 100).

*8-Carboxylic acid-2-ethoxy-4H-[1,2,4]triazolo[[1,5-a]]quinazolin-5-one* (**5b**). IR (cm^−1^): ν 1,689, 1,703 (C=O). ^1^H-NMR (DMSO-d_6_): δ 1.38 (t, *J* = 7.02 Hz, 3H, OCH_2_C*H_3_*), 3.34 (s, 1H, OH), 4.35 (q, *J* = 14.10 Hz, 2H, OC*H_2_*CH_3_), 7.67-8.12 (m, 3H, Ar-H), 13.01 (s, 1H, NH).^ 13^C-NMR: 14.86, 65.64, 114.29, 116.79, 125.43, 128.25, 135.62, 136.12, 147.24, 156.14, 159.86, 167.52. MS, *m/z* (%): 274 (M^+^, 95). 

*8-Methyl-2-pentyloxy-4H-[1,2,4]triazolo[1,5-a]quinazolin-5-one* (**5c**). IR (cm^−1^): ν 1,690 (C=O). ^1^H-NMR (DMSO-d_6_): δ 0.98 (t, *J* = 7.32 Hz, 3H, OCH_2_CH_2_CH_2_CH_2_C*H_3_*), 1.37-1.44 (m, 4H, OCH_2_CH_2_C*H_2_*C*H_2_*CH_3_), 1.63-1.79 (m, 2H, OCH_2_C*H_2_*CH_2_CH_2_CH_3_), 2.78 (s, 3H, CH_3_), 4.42 (t, *J* = 7.41 Hz, 2H, OC*H_2_*CH_2_CH_2_CH_2_CH_3_), 7.45-8.51 (m, 3H, Ar-H), 12.83 (s, 1H, NH). ^13^C-NMR: 13.39, 14.47, 22.24, 27.73, 28.07, 69.74, 114.65, 116.80, 125.45, 128.68, 135.72, 136.11, 147.74, 159.91, 167.70. MS, *m/z* (%): 286 (M^+^, 85).

*2-Allyloxy-8-methyl-4H-[1,2,4]triazolo[1,5-a]quinazolin-5-one* (**5d**). IR (cm^−1^): ν 1,697 (C=O). ^1^H-NMR (DMSO-d_6_): δ 3.39 (s, 3H, CH_3_), 4.86 (d, *J* = 5.68 Hz, 2H, CH_2_=CHC*H_2_*), 5.42-5.61 (m, 2H, C*H_2_*=CHCH_2_), 6.05-6.15 (m, 1H, CH_2_=C*H*CH_2_), 7.68-8.25 (m, 3H, Ar-H), 13.41 (s, 1H, NH). ^13^C-NMR: 23.89, 69.60, 113.82, 116.44, 118.25, 125.16, 128.13, 134.11, 135.30, 135.62, 147.30, 159.45, 166.92. MS, *m/z* (%): 256 (M^+^, 100). 

*2-Benzyloxy-7,8-dimethoxy-4H-[1,2,4]triazolo[1,5-a]quinazolin-5-one* (**5e **IR (cm^−1^): ν 1,710 (C=O). ^1^H-NMR (DMSO-d_6_): δ 3.48 (s, 3H, OCH_3_), 3.98 (s, 3H, OCH_3_), 5.39 (s, 2H, OC*H_2_*Ph), 7.37-8.16 (m, 7H, Ar-H), 13.44 (s, 1H, NH). ^13^C-NMR: 54.23, 58.09, 71.18, 114.34, 116.81, 125.53, 127.74, 128.03, 128.85, 135.75, 136.11, 136.77, 147.11, 160.40, 167.58. MS, *m/z* (%): 352 (M^+^, 92). 

*7,8-Dimethoxy-2-phenethyloxy-4H-[1,2,4]triazolo[1,5-a]quinazolin-5-one* (**5f**). IR (cm^−1^): ν 1,689 (C=O). ^1^H-NMR (DMSO-d_6_): δ 3.09 (t, *J* = 7.44 Hz, 2H, OC*H_2_*CH_2_Ph), 3.80 (s, 3H, OCH_3_), 4.01 (s, 3H, OCH_3_), 4.50 (t, *J* = 7.41 Hz, 2H, OCH_2_C*H_2_*Ph), 7.20-8.19 (m, 7H, Ar-H), 13.75 (s, 1H, NH). ^13^C-NMR: 34.91, 51.73, 56.71, 70.23, 116.81, 114.32, 126.80, 125.51, 128.29, 128.74, 129.37, 136.14, 138.33, 147.72, 159.91, 167.57. MS, *m/z* (%): 366 (M^+^, 53). 

*8-Methyl-2-methylsulfanyl-4H-[1,2,4]triazolo[1,5-a]quinazolin-5-one* (**5g**). IR (cm^−1^): ν 1,687 (C=O). ^1^H-NMR (DMSO-d_6_): δ 2.94 (s, 3H, CH_3_), 3.27 (s, 3H, SCH_3_) 7.64-8.25 (m, 3H, Ar-H), 13.68 (s, 1H, NH). ^13^C-NMR: 13.92, 24.60, 114.65, 116.23, 125.50, 128.58, 135.72, 136.12, 149.11, 159.90, 162.30. MS, *m/z* (%): 246 (M^+^, 87). 

*7,8-Dimethoxy-2-methylsulfanyl-4H-[1,2,4]triazolo[1,5-a]quinazolin-5-one* (**5h**). IR (cm^−1^): ν 1,698 (C=O). ^1^H-NMR (DMSO-d_6_): δ 2.87 (s, 3H, SCH_3_), 3.07 (s, 3H, OCH_3_), 3.84 (s, 3H, OCH_3_), 7.59-8.36 (m, 2H, Ar-H), 13.90 (s, 1H, NH). ^13^C-NMR: 13.78, 56.45, 58.01, 114.05, 115.91, 126.34, 129.08, 135.09, 136.52, 149.11, 159.72, 165.30. MS, *m/z* (%): 292 (M^+^, 100). 

#### 3.2.2. Synthesis of compounds **6a-d**

To a solution of **5** (1 mmol) in DMF (5 mL) was added potassium carbonate (1.2 mmol) portion wise over a period of 10 min at room temperature. After stirring for 20 min, the appropriate alkyl halide (1.5 mmol) was added dropwise and the reaction mixture was stirred for 18 h at room temperature. The mixture was poured into ice/water, the precipitate was filtered off, washed with water and dried. Analytically pure products **6a-d** were obtained after recrystallization from THF.

*8-Methyl-2-methylsulfanyl-4-phenethyl[1,2,4]triazolo[1,5-a]quinazolin-5-one* (**6a**). IR (cm^−1^): ν 1,671 (C=O). ^1^H-NMR (DMSO-d_6_): δ 2.98 (s, 3H, SCH_3_), 3.37 (t, *J* = 7.54 Hz, 2H, NC*H_2_*CH_2_Ph), 4.02 (s, 3H, CH_3_), 4.31 (t, *J* = 7.51 Hz, 2H, NCH_2_C*H_2_*Ph), 7.22-8.20 (m, 8H, Ar-H). ^13^C-NMR: 13.98, 24.74, 34.48, 64.26, 114.68, 116.13, 125.82, 126.88, 128.87, 135.42, 135.84, 138.39, 147.59, 158.76, 167.91. MS, *m/z* (%): 350 (M^+^, 90). 

*2-Benzyloxy-7,8-dimethoxy-4-allyl[1,2,4]triazolo[1,5-a]quinazolin-5-one* (**6b**). IR (cm^−1^): ν 1,678 (C=O). ^1^H-NMR (DMSO-d_6_): δ 3.11 (s, 3H, OCH_3_), 3.90 (s, 3H, OCH_3_), 4.45 (d, *J* = 5.62 Hz, 2H, CH_2_=CHC*H_2_*), 5.17-5.29 (m, 2H, C*H_2_*=CHCH_2_), 5.31 (s, 2H, CH_2_), 6.25-6.33 (m, 1H, CH_2_=C*H*CH_2_), 7.50-8.30 (m, 7H, Ar-H). ^13^C-NMR: 24.11, 49.89, 57.34, 69.60, 113.82, 116.44, 118.25, 124.03, 124.98, 125.16, 128.13, 131.54, 134.91, 135.30, 135.62, 147.30, 159.05, 165.82. MS, *m/z* (%): 392 (M^+^, 79). 

*8-Carboxylic acid-4-ethyl-2-methoxy[1,2,4]triazolo[1,5-a]quinazolin-5-one* (**6c**). IR (cm^−1^): ν 1,675, 1,682 (C=O). ^1^H-NMR (DMSO-d_6_): δ 1.37 (t, *J* = 7.02 Hz, 3H, NCH_2_*CH_3_*), 3.52 (s, 1H, OH), 4.09 (q, *J* =14.22 Hz, 2H, NC*H_2_*CH_3_), 4.32 (s, 3H, OCH_3_), 7.58-8.09 (m, 3H, Ar-H). ^13^C-NMR: 14.23, 52.34, 57.18, 114.13, 116.27, 125.71, 128.79, 135.20, 135.42, 148.49, 158.64, 162.34, 167.99. MS, *m/z* (%): 288 (M^+^, 67). 

*2-Allyloxy-4-benzyl-8-methyl[1,2,4]triazolo[1,5-a]quinazolin-5-one* (**6d**). IR (cm^−1^): ν 1,670 (C=O). ^1^H-NMR (DMSO-d_6_): δ 3.57 (s, 3H, CH_3_), 4.83 (d, *J* = 4.60 Hz, 2H, CH_2_=CHC*H_2_*), 5.20 (s, 2H, CH_2_), 5.30-5.42 (m, 2H, C*H_2_*=CHCH_2_), 6.04-6.14 (m, 1H, CH_2_=C*H*CH_2_), 7.46-8.15 (m, 8H, Ar-H). ^13^C-NMR: 24.29, 44.53, 63.14, 114.47, 116.10, 117.18, 125.83, 128.82, 130.23, 131.66, 134.45, 134.90, 135.32, 135.86, 148.79, 157.81, 168.43. MS, *m/z* (%): 346 (M^+^, 80). 

#### 3.2.3. Synthesis of compounds **7a-d**

A solution of **5 (**1 mmol**) **in dry THF (5 mL) was added dropwise to a stirred suspension of LiAlH_4_ (3 mmol) in dry THF (10 mL). After stirring at room temperature for 3 h, water (0.4 mL) was added carefully and the mixture was stirred for an additional 30 min. The reaction mixture was filtered and the solvent removed under reduced pressure, the residue was dissolved in THF and passed through a short column chromatography, the solvent was removed under reduced pressure, and the obtained solid was recrystallized from EtOAc/*n*-hexane.

*4,5-Dihydro-8-methyl-2-methylsulanyl[1,2,4]triazolo[1,5-a]quinazoline* (**7a**). IR (cm^−1^): ν 3,167, (NH). ^1^H-NMR (DMSO-d_6_): δ 2.83 (s, 3H, CH_3_), 3.50 (s, 3H, SCH_3_), 4.20 (s, 2H, CH_2_-quinazoline), 7.28-7.82 (m, 3H, Ar-H), 7.95 (s, 1H, NH). ^13^C-NMR: 13.23, 25.23, 43.22, 112.72, 119.64, 124.50, 126.23, 130.75, 134.16, 155.18, 165.29. MS, *m/z* (%): 232 (M^+^, 100).

*2-Allyloxy-4,5-dihydro-8-methyl[1,2,4]triazolo[1,5-a]quinazoline* (**7b**). IR (cm^-1^): ν 3,153, (NH). ^1^H-NMR (DMSO-d_6_): δ 2.76 (s, 3H, CH_3_), 4.76 (d, *J* = 6.74 Hz, 2H, CH_2_=CHC*H_2_*), 4.92 (s, 2H, CH_2_-quinazoline), 5.22-5.33 (m, 2H, C*H_2_*=CHCH_2_), 6.09-6.16 (m, 1H, CH_2_=C*H*CH_2_), 7.48-8.10 (m, 3H, Ar-H), 8.25,(s, 1H, NH).^ 13^C-NMR: 23.45, 69.63, 113.87, 116.45, 118.20, 119.24, 125.33, 128.12, 134.57, 135.25, 135.51, 159.47, 167.70. MS, *m/z* (%): 242 (M^+^, 89).

*2-Benzyloxy-4,5-dihydro-7,8-dimethoxy[1,2,4]triazolo[1,5-a]quinazoline* (**7c**). IR (cm^−1^): ν 3,189, (NH). ^1^H-NMR (DMSO-d_6_): δ 2.93 (s, 3H, OCH_3_), 3.30 (s, 3H, OCH_3_), 4.50 (s, 2H, CH_2_-quinazoline), 5.26 (s, 2H, OC*H_2_*Ph), 7.01-7.56 (m, 7H, Ar-H), 7.91 (s, 1H, NH). ^13^C-NMR: 52.07, 55.39, 69.94, 112.27, 119.23, 124.10, 126.30, 127.12, 127.75, 128.23, 128.85, 133.27, 136.44, 154.55, 166.87. MS, *m/z* (%): 338 (M^+^, 93).

*4,5-Dihydro-7,8-dimethoxy-2-phenethyloxy[1,2,4]triazolo[1,5-a]quinazoline* (**7d**). IR (cm^−1^): ν 3,180, (NH). ****^1^H-NMR (DMSO-d_6_): δ 2.89 (s, 3H, OCH_3_), 3.2 2 (s, 3H, OCH_3_), 3.44 (t, *J* = 7.45 Hz, 2H, OC*H_2_*CH_2_Ph), 4.39 (t, *J* = 7.41 Hz, 2H, OCH_2_C*H_2_*Ph), 4.48 (s, 2H, CH_2_-quinazoline), 7.10-7.52 (m, 7H, Ar-H), 7.77 (s, 1H, NH). ^13^C-NMR: 34.90, 45.07, 49.38, 68.95, 113.33, 119.27, 124.23, 126.80, 128.51, 129.37, 131.09, 135.74, 136.11, 138.33, 154.90, 166.85. MS, *m/z* (%): 352 (M^+^, 90). 

#### 3.2.4. Synthesis of compounds **8a-d**

Compound **5** (1 mmol) was refluxed with phosphorus pentasulfide (1 mmol) in absolute pyridine (5 mL) for 2 h. Afterwards the reaction mixture was cooled and poured into ice/water, the yellow precipitate was separated by filtration and washed thoroughly with water. Recrystallization from aqueous DMF furnished analytically pure **8a-d**. 

*7,8-Dimethoxy-2-methylsulfanyl-4H-[1,2,4]triazolo[1,5-a]quinazolin-5-thione* (**8a**). IR (cm^−1^): ν 1,268 (C=S). ^1^H-NMR (DMSO-d_6_): δ 3.32 (s, 3H, SCH_3_), 3.70 (s, 3H, OCH_3_), 4.02 (s, 3H, OCH_3_), 7.52-7.96 (m, 2H, Ar-H), 14.72 (s, 1H, NH). ^13^C-NMR: 13.72, 54.43, 56.84, 114.21, 122.43, 125.83, 132.41, 135.88, 149.59, 162.78, 185.71. MS, *m/z* (%): 308 (M^+^, 100). 

*2-Allyloxy-8-methyl-4H-[1,2,4]triazolo[1,5-a]quinazolin-5-thione*** (8b**). IR (cm^−1^): ν 1,258 (C=S). ^1^H-NMR (DMSO-d_6_): δ 2.86 (s, 3H, CH_3_), 4.85 (d, *J* = 6.36 Hz, 2H, CH_2_=CHC*H_2_*), 5.31-5.46 (m, 2H, C*H_2_*=CHCH_2_), 6.08-6.15 (m, 1H, CH_2_=C*H*CH_2_), 7.48-8.12 (m, 3H, Ar-H), 14.48 (s, 1H, NH). ^13^C-NMR: 25.09, 69.92, 114.27, 118.39, 122.53, 125.92, 128.21, 131.83, 132.42 , 135.92, 145.75, 167.31, 184.91. MS, *m/z* (%): 272 (M^+^, 94). 

*2-Benzyloxy-7,8-dimethoxy-4H-[1,2,4]triazolo[1,5-a]quinazolin-5-thione* (**8c**). IR (cm^−1^): ν 1,255 (C=S). ^1^H-NMR (DMSO-d_6_): δ 3.20 (s, 3H, OCH_3_), 3.78 (s, 3H, OCH_3_), 5.42 (s, 2H, OC*H_2_*Ph), 7.37-8.62 (m, 7H, Ar-H), 14.74 (s, 1H, NH). ^13^C-NMR: 45.21, 48.34, 70.60, 114.24, 122.40, 125.37, 128.06, 128.30, 128.90, 131.72, 132.33, 135.38, 145.90, 167.34, 185.11. MS, *m/z* (%): 368 (M^+^, 65).

*7,8-Dimethoxy-2-phenethyloxy-4H-[1,2,4]triazolo[1,5-a]quinazolin-5-thione* (**8d**). IR (cm^−1^): ν 1,249 (C=S). ^1^H-NMR (DMSO-d_6_): δ 2.95 (s, 3H, OCH_3_), 3.11 (t, *J* = 6.35 Hz, 2H, OC*H_2_*CH_2_Ph), 3.58 (s, 3H, OCH_3_), 4.55 (t, *J* = 6.63 Hz, 2H, OCH_2_C*H_2_*Ph), 7.24-8.61 (m, 7H, Ar-H), 14.70 (s, 1H, NH). ^13^C-NMR: 34.45, 47.21, 53.34, 69.72, 114.23, 122.42, 125.00, 125.82, 126.30, 128.85, 131.76, 135.83, 137.80, 145.63, 165.20, 186.62. MS, *m/z* (%): 382 (M^+^, 78).

#### 3.2.5. Synthesis of compounds **9a-f**

**Method-A: **Compound **5** (2 mmol) was refluxed with oxalyl chloride (6 mmol) in 1,1,2-trichloroethane (12 mL) for 19 h at 105°C. The solution was cooled and MeOH (0.2 mL) was added drop- wise, the obtained solid was filtered, washed with hexane, dried and recrystallized from THF-hexane.

**Method-B:** Compound **5** (1 mmol) was refluxed with Phosphorus oxychloride (1 mL) in benzene (7 mL) for 2 h. The solvent was evaporated and the residue was treated with saturated aqueous solution of potassium carbonate. The solid was filtered, washed thoroughly with water, dried and recrystallized from THF-hexane.

*8-Carboxylic acid-5-chloro-2-ethoxy[1,2,4]triazolo[1,5-a]quinazoline* (**9a). **IR (cm^−1^): ν 1,683 (C=O). ^1^H-NMR (DMSO-d_6_): δ 1.30 (t, *J* = 7.07 Hz, 3H, OCH_2_C*H_3_*), 3.03 (s, 1H, OH), 4.34 (q, *J* = 14.10 Hz, 2H, OC*H_2_*CH_3_), 7.49-8.15 (m, 3H, Ar-H). ^13^C-NMR: 14.56, 64.57, 114.38, 116.05, 125.39, 128.22, 135.58, 136.43, 142.70, 154.32, 159.38. MS, *m/z* (%): 292 (M^+^, 88). 

*5-Chloro-8-methyl-2-pentyloxy[1,2,4]triazolo[1,5-a]quinazoline* **(9b). **^1^H-NMR (DMSO-d_6_): δ 0.81 (t, *J* = 7.45 Hz, 3H, OCH_2_CH_2_CH_2_CH_2_C*H_3_*), 1.46-1.63 (m, 4H, OCH_2_CH_2_C*H_2_*C*H_2_*CH_3_), 1.83-1.89 (m, 2H, OCH_2_C*H_2_*CH_2_CH_2_CH_3_), 3.11 (s, 3H, CH_3_), 4.43 (t, *J* = 7.60 Hz, 2H, OC*H_2_*CH_2_CH_2_CH_2_CH_3_), 7.45-8.16 (m, 3H, Ar-H). ^13^C-NMR: 13.75, 21.70, 27.35, 28.16, 45.82, 69.52, 114.70, 116.81, 126.54, 127.95, 136.57, 146.63, 155.33, 160.07. MS, *m/z* (%): 304 (M^+^, 91).

*2-Benzyloxy-5-chloro-7,8-dimethoxy[1,2,4]triazolo[1,5-a]quinazoline*** (9c).** ^1^H-NMR (DMSO-d_6_): δ 3.23 (s, 3H, OCH_3_), 3.64 (s, 3H, OCH_3_), 5.79 (s, 2H, OC*H_2_*Ph), 7.37-8.45 (m, 7H, Ar-H). ^13^C-NMR: 50.01, 53.74, 71.34, 115.20, 117.42, 125.50, 126.71, 127.14, 128.25, 128.70, 132.41, 135.90, 136.11, 136.77, 155.93, 162.65. MS, *m/z* (%): 370 (M^+^, 69).

*2-Allyloxy-5-chloro-8-methyl[1,2,4]triazolo[1,5-a]quinazoline*** (9d). **^1^H-NMR (DMSO-d_6_): δ 3.01 (s, 3H, CH_3_), 4.21 (d, *J* = 5.50 Hz, 2H, CH_2_=CHC*H_2_*), 5.33-5.52 (m, 2H, C*H_2_*=CHCH_2_), 6.10-6.16 (m, 1H, CH_2_=C*H*CH_2_), 7.73-8.34 (m, 3H, Ar-H). ^13^C-NMR: 25.89, 69.60, 113.82, 116.44, 118.25, 125.16, 128.13, 134.11, 135.30, 135.62, 145.87, 158.33, 163.12. MS, *m/z* (%): 274 (M^+^, 100). 

*5-Chloro-7,8-dimethoxy-2-phenethyloxy[1,2,4]triazolo[1,5-a]quinazoline*** (9e): **^1^H-NMR (DMSO-d_6_): δ 3.09 (s, 3H, OCH_3_), 3.21 (t, *J* = 7.50 Hz, 2H, OC*H_2_*CH_2_Ph), 3.52 (s, 3H, OCH_3_), 4.65 (t, *J* = 7.51 Hz, 2H, OCH_2_C*H_2_*Ph), 7.22-8.37 (m, 7H, Ar-H). ^13^C-NMR: 35.09, 49.60, 54.11, 69.61, 114.59, 124.40, 124.83, 126.72, 128.74, 129.30, 134.29, 134.94, 138.49, 153.37, 156.84, 161.31. MS, *m/z* (%): 384 (M^+^, 100).

*5-Chloro-8-methyl-2-methylsulfanyl[1,2,4]triazolo[1,5-a]quinazoline*** (9f).**^ 1^H-NMR (DMSO-d_6_): δ 3.12 (s, 3H, CH_3_), 3.72 (s, 3H, SCH_3_) 7.34-8.15 (m, 3H, Ar-H). ^13^C-NMR: 13.62, 24.00, 115.15, 117.83, 125.36, 128.18, 136.02, 136.92, 149.80, 152.24, 158.93. MS, *m/z* (%): 264 (M^+^, 93).

#### 3.2.6. Synthesis of compounds **10a-d**

Compound **9 **(1 mmol) was heated under reflux with hydrazine hydrate (5 mmol) in EtOH (8 mL) for 3 h. After cooling, the precipitate was filtered off and washted with water. Recrystallization from EtOH afforded **10a-d** as colored pure solids.

*8-Methyl-2-methylsulfanyl[1,2,4]triazolo[[1,5-a]]quinazolin-5-yl-hydrazine* (**10a**). IR (cm^−1^): ν 3,189, 3,231 (NH-NH_2_). ^1^H-NMR (DMSO-d_6_): δ 2.80 (s, 3H, CH_3_), 3.78 (s, 3H, SCH_3_), 4.84 (s, 2H, NH_2_), 7.97-8.30 (m, 3H, Ar-H), 9.37 (s, 1H, NH). ^13^C-NMR: 13.87, 26.48, 114.48, 124.06, 124.77, 125.40, 127.35, 134.24, 134.88, 153.31, 169.73. MS, *m/z* (%): 260 (M^+^, 100).

*8-Carboxylic acid-2-ethoxy[1,2,4]triazolo[[1,5-a]]quinazolin-5-yl-hydrazine* (**10b**). IR (cm^−1^): ν 3,182, 3,201 (NH-NH_2_), 1,686 (C=O). ^1^H-NMR (DMSO-d_6_): δ 1.32 (t, *J* = 7.07 Hz, 3H, OCH_2_*CH_3_*), 3.34 (s, 1H, OH), 4.37 (q, *J* = 14.15 Hz, 2H, OC*H_2_C*H_3_), 4.65 (s, 2H, NH_2_), 7.89-8.05 (m, 3H, Ar-H), 9.42 (s, 1H, NH). ^13^C-NMR: 14.73, 65.61, 114.12, 116.45, 125.62, 128.43, 135.13, 136.29, 145.38, 157.82, 167.92. MS, *m/z* (%): 288 (M^+^, 78). 

*2-Allyloxy-8-methyl[1,2,4]triazolo[[1,5-a]]quinazolin-5-yl-hydrazine* (**10c**). IR (cm^−1^): ν 3,210, 3,267 (NH-NH_2_). ^1^H-NMR (DMSO-d_6_): δ 3.21 (s, 3H, CH_3_), 4.81 (s, 2H, NH_2_), 4.85 (d, *J* = 5.30 Hz, 2H, CH_2_=CHC*H_2_*), 5.29-5.43 (m, 2H, C*H_2_*=CHCH_2_), 6.06-6.12 (m, 1H, CH_2_=C*H*CH_2_) 7.87-8.31 (m, 3H, Ar-H), 9.90 (s, 1H, NH). ^13^C-NMR: 25.34, 69.53, 70.55, 113.12, 114.59, 118.32, 124.41, 133.52, 134.26, 134.95, 150.72, 161.12, 168.50. MS, *m/z* (%): 270 (M^+^, 94). 

*2-Benzyloxy-7,8-dimethoxy[1,2,4]triazolo[[1,5-a]]quinazolin-5-yl-hydrazine* (**10d**). IR (cm^−1^): ν 3,205, 3,286 (NH-NH_2_). ^1^H-NMR (DMSO-d_6_): δ 3.08 (s, 3H, OCH_3_), 3.43 (s, 3H, OCH_3_), 4.82 (s, 2H, OC*H_2_*Ph), 5.40 (s, 2H, NH_2_), 7.93-8.32 (m, 7H, Ar-H), 9.84 (s, 1H, NH). ^13^C-NMR: 43.21, 47.87, 69.63, 113.85, 116.43, 118.20, 125.12, 128.15, 132.62, 135.15, 135.66, 147.34, 159.45, 166.93. MS, *m/z* (%): 366 (M^+^, 72). 

#### 3.2.7. Synthesis of compounds **11a-d**

A mixture of **10 **(1 mmol**) **and aldehyde or ketone (1 mmol) was refluxed in EtOH (10 mL) for 3 h. The solvent was removed under reduced pressure, and the resulting solids were recrystallized from EtOH. 

*N-Isopropylidene-N^'^-(8-methyl-2-methylsulfanyl[1,2,4]triazolo[[1,5-a]]quinazolin-5-yl)hydrazine* (**11a**). ^1^H-NMR (DMSO-d_6_): δ 2.21 (s, 3H, CH_3_-isopropyl), 2.63 (s, 3H, CH_3_-isopropyl), 2.85 (s, 3H, CH_3_), 3.45 (s, 3H, SCH_3_), 7.37-7.94 (m, 3H, Ar-H), 10.45 (s, 1H, NH). ^13^C-NMR: 13.80, 18.67, 25.27, 45.34, 115.08, 124.90, 125.75, 126.06, 134.24, 134.96, 162.37, 164.54. MS, *m/z* (%): 300 (M^+^, 79). 

*N-Benzylidene-N^'^-(8-methy-2-methylsulfanyl[1,2,4]triazolo[[1,5-a]]-quinazolin-5-yl)hydrazine* (**11b**). ^1^H-NMR (DMSO-d_6_): δ 2.92 (s, 3H, CH_3_), 3.34 (s, 3H, SCH_3_), 4.33 (s, 1H, CH-benzylidene), 7.45-8.05 (m, 8H, Ar-H), 11.83 (s, 1H, NH). ^13^C-NMR: 13.78, 25.17, 69.79, 110.23, 114.12, 115.37, 124.65, 127.54, 129.20, 131.76, 133.54, 141.32, 154.20, 168.97. MS, *m/z* (%): 348 (M^+^, 90). 

*N-(2-Benzyloxy-7,8-dimethoxy[1,2,4]triazolo[1,5-a]quinazolin-5-yl)-N^'^-isopropylidene-hydrazine* (**11c**). ^1^H-NMR (DMSO-d_6_): δ 2.12 (s, 3H, CH_3_-isopropyl), 2.30 (s, 3H, CH_3_-isopropyl ), 2.87 (s, 3H, OCH_3_), 3.33 (s, 3H, OCH_3_), 5.54 (s, 2H, OC*H_2_*Ph), 6.34 (s, 1H, NH), 7.37-8.63 (m, 7H, Ar-H). ^13^C-NMR: 12.71, 13.43, 52.65, 58.43, 70.88, 103.16, 109.37, 114.05, 121.53, 124.69, 128.18, 128.76, 136.08, 136.74, 143.30, 150.85, 152.63, 169.21. MS, *m/z* (%): 406 (M^+^, 80). 

*N-(2-Phenethyloxy)-7,8-dimethoxy[1,2,4]triazolo[1,5-a]quinazolin-5-yl)-N^'^-(1-phenyl-ethylidene)-hydrazine* (**11d**). ^1^H-NMR (DMSO-d_6_): δ 2.56 (s, 3H, OCH_3_), 3.17 (t, *J* = 7.74 Hz, 2H, OC*H_2_*CH_2_Ph), 3.34 (s, 3Η, CH_3_-ethylidene), 3.83 (s, 3H, OCH_3_), 4.77 (t, *J* = 7.71 Hz, 2H, OCH_2_C*H_2_*Ph), 7.25-8.55 (m, 12H, Ar-H), 9.91 (s, 1H, NH). ^13^C-NMR: 14.65, 35.10, 51.11, 56.43, 69.79, 110.73, 114.62, 116.73, 124.65, 125.33, 126.23, 128.11, 129.20, 131.11, 131.58, 132.27, 135.78, 139.52, 141.32, 145.34, 152.20, 161.57. MS, *m/z* (%): 482 (M^+^, 64). 

#### 3.2.8. Synthesis of compounds **12a,b**

A mixture of **10 **(0.5 mmol) and 1,1^'^-carbonyldiimidazole (0.6 mmol) was refluxed in absolute toluene (7 mL) for 3 h. The solvent was removed under reduced pressure and the residue was treated with CHCl_3_. The resulting solid was separated by filtration and recrystallized from EtOH.

*2-Allyloxy-8-methyl-bis[1,2,4]triazolo[1,5-a:4',3'-c]quinazolin-3-one* (**12a**). IR (cm^−1^): ν 1,702 (C=O). ^1^H-NMR (DMSO-d_6_): δ 3.01 (s, 3H, CH_3_), 4.60 (d, *J* = 5.54 Hz, 2H, CH_2_=CHC*H_2_*), 5.20-5.39 (m, 2H, C*H_2_*=CHCH_2_), 6.10-6.18 (m, 1H, CH_2_=C*H*CH_2_), 7.31-7.92 (m, 3H, Ar-H), 12.24 (s, 1H, NH). ^13^C-NMR: 24.74, 65.09, 114.92, 125.12, 127.64, 129.03, 131.23, 135.66, 136.76, 148.73, 157.43, 168.37. MS, *m/z* (%): 296 (M^+^, 75). 

*2-Benzyloxy-7,8-dimethoxy-bis[1,2,4]triazolo[1,5-a:4',3'-c]quinazolin-3-one* (**12b**). IR (cm^−1^): ν 1,711 (C=O). ^1^H-NMR (DMSO-d_6_): δ 3.75 (s, 3H, OCH_3_), 4.03 (s, 3H, OCH_3_), 5.41 (s, 2H, OC*H_2_*Ph), 7.38-8.22 (m, 7H, Ar-H), 12.87 (s, 1H, NH). ^13^C-NMR: 54.76, 56.65, 70.53, 110.38, 114.59, 120.67, 124.87, 128.48, 133.33, 134.30, 134.89, 136.89, 147.67, 153.38, 156.80, 168.62. MS, *m/z* (%): 392 (M^+^, 82). 

#### 3.2.9. Synthesis of compounds **13a,b**

A mixture of **10** (0.5 mmol) and CS_2_ (2.5 mmol) in pyridine (5 mL) was refluxed for 2 h. After cooling, the mixture was poured into ice/water, the yellow precipitate was filtered off, washed with water and recrystallized from MeOH.

*2-Benzyloxy-7,8-dimethoxy-bis[1,2,4]triazolo[1,5-a:4',3'-c]quinazolin-3-thione* (**13a**). ^1^H-NMR (DMSO-d_6_): δ 3.88 (s, 3H, OCH_3_), 4.43 (s, 3H, OCH_3_), 5.11 (s, 2H, OC*H_2_*Ph), 7.43-8.18 (m, 7H, Ar-H), 14.60 (s, 1H, NH). ^13^C-NMR: 49.06, 55.78, 71.52, 112.05, 115.19, 124.16, 125.63, 126.87, 128.34, 128.83, 133.33, 134.42, 136.15, 142.06, 157.12, 163.17, 185.73. MS, *m/z* (%): 408 (M^+^, 90).

*8-Methyl-2-methylsulfanyl-bis[1,2,4]triazolo[1,5-a:4',3'-c]quinazolin-3-thione* (**13b**). ^1^H-NMR (DMSO-d_6_): δ 2.82 (s, 3H, CH_3_), 3.90 (s, 3H, SCH_3_) 7.74-8.15 (m, 3H, Ar-H), 14.68 (s, 1H, NH). ^13^C-NMR: 13.72, 24.60, 114.75, 115.23, 126.57, 129.53, 1350, 136.32, 148.91, 159.94, 162.30, 185.05. MS, *m/z* (%): 302 (M^+^, 83). 

#### 3.2.10. Synthesis of compounds **14a,b**

A mixture of **9 **(1 mmol) and the corresponding carbohydrazide (2.2 mmol) was refluxed in toluene (10 mL) for 2.5 h. After cooling, the solid was collected by filtration. Analytically pure products **14a**,**b** were obtained by recrystallization from MeOH.

*8-Methyl-N-(2-methylsulfanyl[1,2,4]triazolo[[1,5-a]]quinazolin-5-yl)-benzohydrazide* (**14a**). IR (cm^−1^): ν 1,660 (C=O), 3,184 (NH). ^1^H-NMR (DMSO-d_6_): δ 2.98 (s, 3H, CH_3_), 4.01(s, 3H, SCH_3_), 7.53-8.51 (m, 8H, Ar-H), 10.39 (s, 1H, NH), 10.97 (s, 1H, NH). ^13^C-NMR: 13.78, 25.67, 109.83, 114.88, 124.93, 125.29, 127.41, 127.99, 128.87, 129.07, 132.21, 132.81, 133.04, 135.09, 157.07, 168.00. MS, *m/z* (%): 364 (M^+^, 92).

*8-Methyl-N-(2-methylsulfanyl[1,2,4]triazolo[[1,5-a]]quinazolin-5-yl)-isonicotinichydrazide* (**14b**). IR (cm^−1^): ν 1,673 (C=O), 3,207 (NH).^ 1^H-NMR (DMSO-d_6_): δ 3.08 (s, 3H, CH_3_), 3.94 (s, 3H, SCH_3_), 7.65-8.50 (m, 7H, Ar-H), 10.66 (s, 1H, NH), 11.14 (s, 1H, NH). ^13^C-NMR: 13.56, 24.69, 109.70, 114.94, 121.73, 124.88, 125.36, 128.45, 133.20, 135.21, 139.74, 150.94, 155.50, 164.82. MS, *m/z* (%): 365 (M^+^, 76).

#### 3.2.11. Synthesis of compounds **15a,b**

A mixture of **9 (**1 mmol) and benzyl carbazate or ethyl carbazate (2.2 mmol) was refluxed in benzene (10 mL) for 2.5 h. The solvent was removed under reduced pressure, the resulting solid was filtered off and recrystallized from MeOH.

*Ethyl-N-(2-allyloxy-8-methyl[1,2,4]triazolo[[1,5-a]]quinazolin-5-yl)-hydrazine-carboxylate* (**15a**). IR (cm^−1^): ν 1708 (C=O), 3198 (NH).^ 1^H-NMR (DMSO-d_6_): δ 1.13 (t, *J* = 7.61 Hz, 3H, OCH_2_C*H_3_*), 2.87 (s, 3H, CH_3_), 4.08 (q, *J* = 10.12 Hz, 2H, OC*H_2_*CH_3_), 4.60 (d, *J* = 5.54 Hz, 2H, CH_2_=CHC*H_2_*), 5.07-5.19 (m, 2H, C*H_2_*=CHCH_2_), 6.06-6.13 (m, 1H, CH_2_=C*H*CH_2_) 7.54-7.93 (m, 3H, Ar-H), 9.50 (s, 1H, NH), 10.34 (s, 1H, NH). ^13^C-NMR: 14.92, 23.89, 57.33, 63.75, 109.26, 114.60, 124.79, 126.21, 127.07, 134.70, 137.15, 156.69, 169.23. MS, *m/z* (%): 342 (M^+^, 80).

*Benzyl-N-(8-methyl-2-methylsulfanyl[1,2,4]triazolo[[1,5-a]]quinazolin-5-yl)-hydrazine-carboxylate* (**15b**). IR (cm^−1^): ν 1718 (C=O), 3261 (NH).^ 1^H-NMR (DMSO-d_6_): δ 3.74 (s, 3H, CH_3_), 4.11 (s, 3H, SCH_3_), 5.35 (s, 2H, OC*H_2_*Ph), 7.25-8.43 (m, 8H, Ar-H), 9.88 (s, 1H, NH), 10.44 (s, 1H, NH). ^13^C-NMR: 13.54, 25.03, 66.48, 109.62, 114.85, 124.75, 125.25, 126.25, 128.54, 128.81, 135.11, 135.33, 136.98, 152.74, 157.10, 168.42. MS, *m/z* (%): 394 (M^+^, 100).

#### 3.2.12. Synthesis of compounds **16a,b**

A mixture of **14** (0.5 mmol) and POCl_3_ (5 mL) was refluxed at 100°C for 2 h. After cooling, the excess of POCl_3 _was removed under reduced pressure and the residue was treated with saturated aqueous solution of K_2_CO_3_ under ice cooling. The resulting solids were collected by filtration and recrystallized from MeOH to afford **16a**,**b** as colored pure products.

*8-Methyl-2-methylsulfanyl-3-phenyl-bis[1,2,4]triazolo[1,5-a:4',3'-c]quinazoline* (**16a**)**. **^1^H-NMR (DMSO-d_6_): δ 3.33 (s, 3H, CH_3_), 3.91 (s, 3H, SCH_3_), 7.74-8.48 (m, 8H, Ar-H). ^13^C-NMR: 13.65, 24.06, 111.94, 115.17, 124.56, 126.12, 127.09, 128.35, 129.80, 130.59, 131.09, 141.82, 143.67, 149.47, 167.34. MS, *m/z* (%): 346 (M^+^, 82). 

*8-Methyl-2-methylsulfanyl-3-pyridyl-bis[1,2,4]triazolo[1,5-a:4',3'-c]quinazoline* (**16b**). ^1^H-NMR (DMSO-d_6_): δ 3.66 (s, 3H, CH_3_), 4.19 (s, 3H, SCH_3_), 7.53-8.41 (m, 7H, Ar-H). ^13^C-NMR: 13.42, 25.03, 111.24, 115.23, 124.39, 125.75, 128.21, 129.33, 131.16, 133.35, 142.52, 145.67, 150.03, 161.24. MS, *m/z* (%): 347 (M^+^, 95).

#### 3.2.13. Synthesis of compounds **17a-c**

A mixture of **9** (1 mmol) and NaN_3_ (1.2 mmol) in absolute DMF (5 mL) was heated at 90 °C in a nitrogen atmosphere for 24 h. After cooling, the reaction mixture was poured into water and saturated with brine solution. The resulting solid was filtered off, dried and recrystallized from MeOH.

*8-Methyl-2-methylsulfanyl-tetrazolo[4,3-c][1,2,4]triazolo[[1,5-a]]quinazoline* (**17a**). ^1^H-NMR (DMSO-d_6_): δ 3.32 (s, 3H, CH_3_), 3.99 (s, 3H, SCH_3_), 7.48-7.95 (m, 3H, Ar-H). ^13^C-NMR: 13.89, 24.67, 114.67, 116.23, 125.81, 128.27, 134.74, 136.49, 145.01, 157.32, 167.54. MS, *m/z* (%): 271 (M^+^, 77).

*2-Benzyloxy-7,8-dimethoxy-tetrazolo[4,3-c][1,2,4]triazolo[[1,5-a]]quinazoline* (**17b**). ^1^H-NMR (DMSO-d_6_): δ 3.48 (s, 3H, OCH_3_), 4.50 (s, 3H, OCH_3_), 5.75 (s, 2H, OC*H_2_*Ph), 7.44-8.28 (m, 7H, Ar-H). ^13^C-NMR: 45.98, 51.72, 71.57, 109.58, 115.08, 125.5, 127.72, 128.23, 130.36, 134.21, 134.53, 135.47, 148.17, 160.43, 167.16. MS, *m/z* (%): 377 (M^+^, 90).

*7,8-Dimethoxy-2-phenethyloxy-tetrazolo[4,3-c][1,2,4]triazolo[[1,5-a]]quinazoline* (**17c**). ^1^H-NMR (DMSO-d_6_): δ 3.42 (t, *J* = 7.50 Hz, 2H, OC*H_2_*CH_2_Ph), 3.62 (s, 3H, OCH_3_), 4.53 (s, 3H, OCH_3_), 4.79 (t, *J* = 7.51 Hz, 2H, OCH_2_C*H_2_*Ph), 7.23-8.67 (m, 7H, Ar-H). ^13^C-NMR: 41.43, 44.76, 64.83, 71.21, 115.57, 124.39, 126.10, 126.87, 127.56, 128.80, 129.35, 130.23, 134.99, 138.27, 142.32, 156.34, 167.54. MS, *m/z* (%): 391 (M^+^, 100).

#### 3.2.14. Synthesis of compounds **18a,b**

A mixture of **9 (**1 mmol) and 3-aminothiophene-2-methylcarboxylic acid ester (2.2 mmol) in absolute dioxane (10 mL) was refluxed in the presence of NaH (0.4 mmol) for 21 h. The solvent was removed under reduced pressure and the residue was treated with water and MeOH. The resulting solid was filtered off and dried.

*8-Methyl-2-pentyloxy-(3H-thieno[3,2-d]pyrimidin-4-one[4,3-c][1,2,4]triazolo[1,5-a]quinazoline* (**18a**). IR (cm^−1^): ν 1,677 (C=O). ^1^H-NMR (DMSO-d_6_): δ 0.73 (t, *J* = 7.54 Hz, 3H, OCH_2_CH_2_CH_2_CH_2_C*H_3_*), 1.14-1.20 (m, 4H, OCH_2_CH_2_C*H_2_*C*H_2_*CH_3_), 1.48-1.67 (m, 2H, OCH_2_C*H_2_*CH_2_CH_2_CH_3_), 2.87 (s, 3H, CH_3_), 4.08 (t, *J* = 7.60 Hz, 2H, OC*H_2_*CH_2_CH_2_CH_2_CH_3_), 6.78-8.35 (m, 5H, Ar-H). ^13^C-NMR: 14.63, 22.18, 24.65, 27.69, 28.63, 69.35, 114.82, 123.71, 124.25, 124.80, 134.33, 134.89, 147.72, 153.60, 167.84. MS, *m/z* (%): 393 (M^+^, 61).

*7,8-Dimethoxy-2-phenethyloxy-(3H-thieno[3,2-d]pyrimidin-4-one[4,3-c][1,2,4]triazolo[1,5-a]-quinazoline* (**18b**). IR (cm^−1^): ν 1,670 (C=O). ^1^H-NMR (DMSO-d_6_): δ 3.67 (s, 3H, OCH_3_), 3.90 (t, *J* = 7.61 Hz, 2H, OC*H_2_*CH_2_Ph), 4.31 (s, 3H, OCH_3_), 4.60 (t, *J* = 7.63 Hz, 2H, OCH_2_C*H_2_*Ph), 6.38-8.15 (m, 9H, Ar-H). ^13^C-NMR: 36.40, 40.61, 54.70, 63.57, 109.08, 116.08, 124.25, 127.02, 128.13, 128.75, 131.36, 131.93, 133.23, 133.91, 134.53, 135.47, 148.17, 160.43, 167.16. MS, *m/z* (%): 473 (M^+^, 70).

## 4. Conclusions

In summary, the obtained [1,2,4]triazolo[1,5-*a*]quinazolin-5-ones **5a-h** have been used successfully as valuable intermediates in the syntheses of various multifunctional heterocyclic systems. The biological properties of the prepared compounds are still under investigation and will be reported elsewhere.

## Supplementary Materials

Supplementary File 1
