# Al-Salahi, R. Synthesis and Reactivity of [1,2,4]Triazolo-annelated Quinazolines. *Molecules* 2010, *15*, 7016–7034

**DOI:** 10.3390/molecules15118143

**Published:** 2010-11-10

**Authors:** Rashad A. Al-Salahi, Detlef Geffken

**Affiliations:** 1 Departmen of Pharmaceutical Chemistry, College of Pharmacy, King Saud University, Riyadh 11451, Saudi Arabia; 2 Institute of Pharmacy, Chemistry Department, University of Hamburg, Bundesstrasse 45, 20146 Hamburg, Germany; E-Mail: geffken@chemie.uni-hamburg.de (D.G.)

The authors wish to make the following corrections to the paper published in *Molecules* [1]: The authorship of the paper is changed to Rashad A. Al-Salahi and Detlef Geffken. The ‘Acknowledgements’ section of the original publication must be taken as deleted. Finally, the ‘Sample Availability’ information is changed to: Compounds 5-18 are available from Detlef Geffken, University of Hamburg.
