# Supplementary material for: Synthesis and Reactivity of [1,2,4]Triazolo-annelated Quinazolines
Source: Molecules. 2010 Oct 12;15(10):7016–34. doi: 10.3390/molecules15107016 (PMC6259257; doi:10.3390/molecules15107016)
Supplement: Supplementary File 1 [file molecules-15-07016-s001.pdf]

Molecules **2010**, *15*, 8155; doi:10.3390/molecules15118155

OPEN ACCESS

molecules

ISSN 1420-3049

www.mdpi.com/journal/molecules

Correction

Al-Salahi, R. Synthesis and Reactivity of [1,2,4]Triazolo-annelated Quinazolines. *Molecules* 2010, *15*, 7016-7034

Rashad A. Al-Salahi [1,*] and Detlef Geffken [2]

[1] Departmen of Pharmaceutical Chemistry, College of Pharmacy, King Saud University, Riyadh 11451, Saudi Arabia

[2] Institute of Pharmacy, Chemistry Department, University of Hamburg, Bundesstrasse 45, 20146 Hamburg, Germany; E-Mail: geffken@chemie.uni-hamburg.de (D.G.)

* Author to whom correspondence should be addressed; E-Mail: salahi76@yahoo.com.

Received: 9 November 2010 / Accepted: 10 November 2010 / Published: 10 November 2010

The authors wish to make the following corrections to the paper published in *Molecules* [1]: The authorship of the paper is changed to Rashad A. Al-Salahi and Detlef Geffken. The 'Acknowledgements' section of the original publication must be taken as deleted. Finally, the 'Sample Availability' information is changed to: Compounds 5-18 are available from Detlef Geffken, University of Hamburg.

References

1. Al-Salahi, R.A. Synthesis and Reactivity of [1,2,4]Triazolo-annelated Quinazolines. *Molecules* **2010**, *15*, 7016-7034.

© 2010 by the authors; licensee MDPI, Basel, Switzerland. This article is an open access article distributed under the terms and conditions of the Creative Commons Attribution license (http://creativecommons.org/licenses/by/3.0/).
